# Nervous Diseases, Insanity and Bacteriology

**Published:** 1890-11

**Authors:** William C. Krauss


### NERVOUS DISEASES, INSANITY, AND BACTERIOLOGY.

Conducted by WILLIAM C. KRAUSS, M. D.

IN *Neurologisches Centralblatt* for May 15th, Dr. Minor, of Moscow, described a process whereby animal tissues, and especially those of the nervous system, may be hardened in from three to five days. Generally it requires from one to four months to harden tissues in Müller's fluid, before they can be cut. He subjects the tissues to the action of the positive pole in the Müller's fluid, and finds that after three to five days they are sufficiently hardened. Examined under the microscope, the sections were found so hardened. If subjected to the action of the negative pole, they become soft and doughy.

ON THE TRANSMISSIBILITY OF TUBERCULOSIS THROUGH MOUTHPIECES OF MUSICAL INSTRUMENTS.—In the *Revue d'Hygiené*, for April, 1890, Dr. Maljean reported a case of tuberculosis, acquired by using a musical instrument formerly the property of a phthisical patient. To prove his assertion, he poured some sterilized water into the trumpet, allowed it to remain ten minutes, and injected two centimeters of the fluid into a guinea pig. After a short interval, the animal became tubercular and died.

In *The Medical Age*, of August 11th, Dr. V. C. Vaughan, of Ann Arbor, concludes an article on The Fundamental Factors in the Causation of the Infectious Diseases, as follows :

The agent which is most potent in the prevention of the infectious disease through the tissues is the blood. Germicidal properties of this fluid have been abundantly demonstrated; and they are not due to any formed elements, but reside, as has been shown by Buchner, in the proteids. Blood wholly freed from corpuscles and with its leucocytes destroyed by alternate freezing and thawing, still retains its germicidal properties. A healthy condition of the blood and lymph is, therefore, an important factor in the prevention of the infectious diseases.

But you are probably ready to ask: "Of what practical importance is all this?"

It has its practical bearings, and, in brief, they may be summed as follows:

1. All toxicogenic germs are dangerous when introduced in the body.

2. Filth may and does breed disease.

3. It is not necessary that milk should contain a specific germ in order to poison a child, or that a water should contain Eberth's bacillus in order that it may induce typhoid fever.

4. Wheresoever man builds for himself a habitation, and poisons the soil about him, and the water which he drinks, with his own excretions, there will be enteric fever.

5. A good digestion and a healthy gastric juice does aid in the prevention of disease.

6. We can still continue to treat consumption by good, generous feeding and constitutional treatment, without the risk of being denounced old-fogyish and unscientific in our practice. Such practice is more scientific and more successful than such fads as the rectal administration of hydrogen sulphide gas and inhalation of hot air, which have been imported in recent days from France and Germany.

Our fathers, who treated tuberculosis with cod-liver oil and recommended out-door exercise, but who carried no gas bags and knew nothing of the Weigert oven, were not such fools after all.

In conclusion, let us state that the germ is only one of the factors in the causation of the infectious diseases. The other factors are to be found in disordered digestion and impoverished blood.

SUCCESSFUL BRAIN GRAFTING.—W. G. Thompson, who has made numerous experiments in transplanting a piece of brain tissue from one side of an animal's brain to another, or from one animal's brain to another, says:

1. There is complete union, through organized connective tissue, of the contiguous portions of the two brains.

2. After seven weeks the cat's brain still maintained enough vitality to be distinctly recognized as brain tissue.

3. Brains of animals of two very different species were thus made to unite.

4. The pias of cat and dog present perfect union as well.

5. There is a sympathetic degeneration of the corresponding convolutions upon the opposite side of the dog's brain. For this curious fact I cannot account. I have never noticed it before, in as many as fifty operations upon this region of the brain of cats and dogs, although I have sometimes seen removal of a part of the occipital region result in extensive softening of the entire hemisphere of the same side. The opposite degeneration in this case may possibly be a mere coincidence ; if so, it is a very unusual and remarkable one. There was no meningitis to favor it.

6. There was descending secondary degeneration of the dog's brain on the side of the graft, as is usual in cases of simple excision of brain cortex ; hence the cat's cortex had not succeeded in acting as a nutrient center for the dog's brain. (The microscopic specimens, showing the line of union of the two brains, were shown to several competent microscopists, who indorsed their appearance as herein described, so that there can be no question of the accuracy of the observation.)

I think the main fact of this experiment — namely, that brain tissue has sufficient vitality to survive for seven weeks the operation of transplantation without wholly losing its identity as brain substance — suggests an interesting field for further research, and I have no doubt that other experimenters will be rewarded by investigating it.— *New York Medical Journal.*

